# Postoperative delirium following cardiac surgery: the incidence, risk factors and outcome

**DOI:** 10.1186/1749-8090-10-S1-A298

**Published:** 2015-12-16

**Authors:** Judita Andrejaitiene, Rimantas Benetis, Edmundas Sirvinskas

**Affiliations:** 1 Institute of Cardiology, Lithuanian University of Health Sciences, Kaunas, LT-50009, Lithuania; 2 Department of Cardiac, Thoracic and Vascular Surgery, Hospital of Lithuanian University of Health Sciences, Kaunas Clinics, Kaunas, LT-50009, Lithuania

## Background/Introduction

Postoperative delirium (POD) is a common and serious complication after cardiac surgery and numerous studies have confirmed this in occurrence from 10% to 60%, patients have an increased risk of developing POD that is associated with poor outcomes.

## Aims/Objectives

The aim of this study was to identify POD incidence, potential risk factors and to evaluate clinical outcome.

## Method

A single-centre cohort of 292 patients undergoing elective cardiac surgery were prospectively enrolled.

## Results

The incidence of POD was 27.74%. The analysis showed that POD prolonged the length of the ICU stay 5.8 (± 2.89) vs 3.86 (± 1.91) days, p < 0.001, patients after POD more frequent was required re-intubation (OR: 13.169; 95% CI 1.456-119.087, p = 0.022) and had had the prolonged length of the postoperative hospital stay >10 days (OR: 2.060; 95% CI 1.226-3.460, p = 0.006). Multivariate analysis remained as an independent predictors for POD: age > 70 yr (OR: 2.227; 95% CI 1.325-3.742, p = 0.003), ejection fraction < 42% (OR: 2.398; 95% CI 1.397-4.117, p = 0.002), length of stay in the hospital before surgery > 6 days (OR: 1.840; 95% CI 1.064-3.180, p = 0.029), combined valve repair and CABG surgery (OR: 2.083; 95% CI 1.153-3.761, p = 0.015), duration of CPB > 86 min (OR: 2.068; 95% CI 1.182-3.618, p = 0.009) and postoperative atrial fibrillation (OR: 2.244; 95% CI 1.158-4.347, p = 0.007).

## Discussion/Conclusion

Our current analysis suggests that POD is a frequent complication and worsen patient outcome following cardiac surgery. Many factors cannot be changed or avoided but some can be modified and it depends from us: if to shorten the length of stay in the hospital before surgery < 6 days, it may reduce the number of patients who develop POD. By the way, a large prospective randomised study in this regard is needed.

